# Monetary, Food, and Social Rewards Induce Similar Pavlovian-to-Instrumental Transfer Effects

**DOI:** 10.3389/fnbeh.2016.00247

**Published:** 2017-01-04

**Authors:** Rea Lehner, Joshua H. Balsters, Andreas Herger, Todd A. Hare, Nicole Wenderoth

**Affiliations:** ^1^Neural Control of Movement Lab, Department of Health Science and Technology, Federal Institute of Technology ZurichZurich, Switzerland; ^2^Neuroscience Center Zurich, Federal Institute of Technology Zurich, University and Balgrist Hospital Zurich, University of ZurichZurich, Switzerland; ^3^Laboratory for Social and Neural Systems Research, Department of Economics, University of ZurichZurich, Switzerland; ^4^Movement Control and Neuroplasticity Research Group, Department of Kinesiology, Biomedical Sciences Group, KU LeuvenLeuven, Belgium

**Keywords:** Pavlovian-to-instrumental transfer, cue-controlled behavior, Becker-DeGroot-Marschak auction, reward type, subjective reward value, effort

## Abstract

Multiple types of reward, such as money, food or social approval, are capable of driving behavior. However, most previous investigations have only focused on one of these reward classes in isolation, as such it is not clear whether different reward classes have a unique influence on instrumental responding or whether the subjective value of the reward, rather than the reward type *per se*, is most important in driving behavior. Here, we investigate behavior using a well-established reward paradigm, Pavlovian-to-instrumental transfer (PIT), and three different reward types: monetary, food and social rewards. The subjective value of each reward type was matched using a modified Becker-DeGroot-Marschak (BDM) auction where subjective reward value was expressed through physical effort using a bimanual grip force task. We measured the influence of reward-associated stimuli on how participants distributed forces between hands when reaching a target effort range on the screen bimanually and on how much time participants spent in this target range. Participants spent significantly more time in the target range (15% ± 2% maximal voluntary contraction) when a stimulus was presented that was associated with a reward used during instrumental conditioning or Pavlovian conditioning compared to a stimulus associated with a neutral outcome (i.e., general PIT). The strength of the PIT effect was modulated by subjective value (i.e., individuals who showed a stronger PIT effect rated the value of rewards more highly), but not by reward type, demonstrating that stimuli of all reward types were able to act as appetitive reinforcers and influenced instrumental responding, when matched to the same subjective reward value. This is the first demonstration that individually matched monetary, food and social rewards are equally effective as appetitive reinforcers in PIT. These findings strengthen the hypotheses that the subjective value is crucial for how much reward-associated stimuli influence behavior.

## Introduction

Our environment consists of numerous stimuli that are capable of predicting many different types of reward. When deciding how to act, it is commonly assumed that these reward-associated stimuli are compared in order to choose the option associated with the highest value. More generally, there is ample evidence that reward-predicting stimuli can consciously or unconsciously exert a strong influence on behavior (Pessiglione et al., 2008; Holmes et al., 2010; Watson et al., 2016). Despite the well-established links between rewards and actions, one important open question is whether different reward types (i.e., monetary, food or social rewards) are equally effective in motivating behavior.

An important issue is whether different reward types use a separate or a shared valuation system in the brain (Lin et al., 2012; Ruff and Fehr, 2014). Some evidence suggests that, for example, social rewards activate the social cognition network whereas higher-order rewards, such as money, are processed in the anterior part of the ventromedial prefrontal cortex (vmPFC; Saxe, 2006; Sescousse et al., 2013; Clithero and Rangel, 2014). Thus, there is some evidence that different reward types are processed in separate dedicated neural circuits. By contrast, other studies focused on the computation of subjective values for different outcomes, which allows the brain to map different reward types on a common scale to guide choices (Plassmann et al., 2007; Hare et al., 2008; Peters and Büchel, 2010; Rangel and Hare, 2010; Levy and Glimcher, 2011; Clithero and Rangel, 2014). The vmPFC, when the value representation is choice-dependent, the posterior cingulate cortex (PCC), when the value representation happens automatically and the ventral striatum seem to be the main brain regions involved in the computation of subjective value across different reward modalities (Lin et al., 2012; Clithero and Rangel, 2014; Grueschow et al., 2015).

The aim of the present study was to test the influence of reward-associated stimuli on instrumental responding in humans using primary (food), secondary (money) and social (smiling individual in a thumbs-up pose) rewards, while accounting for subjective differences in the valuation of each reward type. Specifically, we tested two alternative hypotheses.

First, we hypothesized that behavior is influenced by reward-associated stimuli independent of reward type, when rewards were individually matched to the same subjective value. Our second competing hypothesis was that stimuli associated with different reward types influence behavior differently, even when the different reward types are equated for subjective value. This might be the case when different reward types use an alternative mechanism to influence instrumental responding or if the reward values are not fully mapped onto a common scale. Furthermore, regardless of whether or not distinct reward types influence behavior differentially, we expected that participants who evaluated the rewards as more valuable (i.e., had a higher subjective value) would show a stronger influence of reward-associated stimuli on behavior.

In order to test our hypotheses, we first measured the subjective values of different reward types such as money, chocolate and a smiling face with a modified Becker-DeGroot-Marschak (BDM) auction using motor effort to individually match the magnitude of monetary and food rewards to the same subjective value as the social reward. These matched rewards were then presented as outcomes during instrumental conditioning, where a response-outcome contingency was learned and Pavlovian conditioning, where a stimulus-outcome contingency was learned. Subsequently, we tested how instrumental responding is influenced by stimuli associated with different reward types under extinction by showing the same stimuli as presented during Pavlovian conditioning in the background meanwhile participants made instrumental responses. This experimental procedure is called Pavlovian-to-instrumental transfer (PIT). The PIT phenomenon has been widely investigated in both animals (for review see Holmes et al., 2010) and humans (Bray et al., 2008; Talmi et al., 2008; Huys et al., 2011, 2016; Prévost et al., 2012; Lewis et al., 2013; Watson et al., 2014; Cartoni et al., 2015; Garofalo and di Pellegrino, 2015; Lovibond et al., 2015; Sebold et al., 2016; Quail et al., 2017) making this a useful model for translational research and in addressing our questions about potential reward-type specific influences on behavior.

We found that the strength of the PIT effect was indeed modulated by subjective value such that individuals who showed a stronger PIT effect rated the value of rewards more highly. However, PIT effects were not systematically different between reward types, demonstrating that monetary, food, and social reward types, if matched on subjective reward value, were equally effective in acting as appetitive reinforcers and in influencing behavior.

## Materials and Methods

### Participants

Sixty-five healthy volunteers (self-reported absence of any physical or psychiatric conditions) were recruited via a university website. All participants gave written informed consent to take part in the experiment. The participants were instructed that the rewards collected during instrumental (depending on their performance) and Pavlovian conditioning will be received at the end of the experiment. The last part of the experiment (PIT test) was conducted under full extinction (i.e., no rewards were available). After completing all aspects of the experiment, all participants were reimbursed at a fixed rate of 20 Swiss Francs per hour and one package of Maltesers^®^ chocolate sweets.

This study was carried out in accordance with the recommendations of the Ethics Committee of the Federal Institute of Technology Zurich with written informed consent from all subjects. All subjects gave written informed consent in accordance with the Declaration of Helsinki (World Medical Association, 2013). We excluded 19 participants because the matching of different reward types was not possible (10), or the Pavlovian conditioning was unsuccessful (3) or they did not follow the instructions (6). The final sample (*N* = 46, *mean age* = 25.34, *standard deviation* = 5.76) consisted of 20 men and 4 left-handers.

### General Procedure

All participants completed a number of questionnaires. Before starting the experiment, participants filled in a handedness questionnaire (Oldfield, 1971) and after the experimental procedure, they answered six multiple choice questions to figure out whether they pursued a conscious strategy during PIT. If they confirmed the first question about using a strategy during PIT, participants were asked to specify their strategy in an open answer. The other five questions offered different strategies and they were told to mark whether or not the strategy was used. In order not to influence the participants’ first answer, we put these questions on a new page. The following strategies were offered: (1) I ignored the fractals (reversed item); (2) If a fractal was shown, which was associated with a reward that I received for either dominant or non-dominant hand use in the first block, I chose to squeeze with the corresponding hand more strongly (corresponds to a specific PIT effect); (3) If a fractal was shown that was previously associated with any of the three rewards, I decided to stay longer in the target (corresponds to a general PIT); (4) If a fractal was shown that was previously associated with my favorite reward, I decided to stay longer in the target; (5) If the fractal was shown that was previously associated with no reward, I decided to not put a lot of effort and to only stay in the target for a short period of time. Based on these six questions, we calculated a total score.

Participants were comfortably seated in a silent room in front of the laptop. The experimental session began with calibrating the grip force handles and measuring the maximal grip force of each hand (mean out of three maximal voluntary contractions; MVC). The required force (50% and 15% of MVC) to reach the target on the screen for the subsequent experimental blocks was computed based on this measurement.

Participants were required to pay attention to the screen and to follow the instructions described at the beginning of each experimental block. Before starting the experiment, several example trials were shown by the experimenter and if needed, further explanations were given.

**Figure 1:**
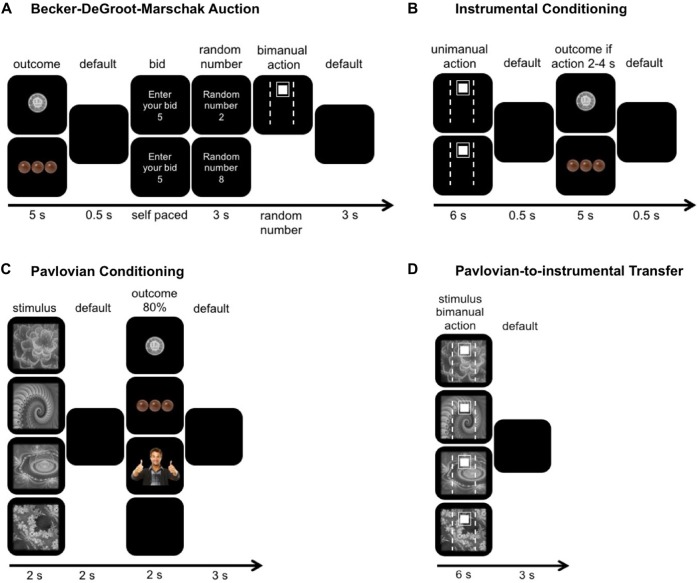
**Graphical illustration of the experimental paradigm. (A)** Participants were presented with the potential outcomes, for example a certain amount of money (upper row) or food (lower row). Participants then bid how many seconds (0–10 s) they would be willing to apply 50% of their maximal grip force in order to receive the displayed reward. If their bid was greater or equal to the random number (upper trial), they had to perform the action which was visualized as the small white filled square going inside the white unfilled rectangle. Otherwise (lower trial) they did not have to do anything but did not win the reward. Lastly, these bids were used to match the monetary and food reward to the subjective value of the social reward. **(B)** During the instrumental conditioning, participants either used their dominant (upper trial) or non-dominant hand (lower trial) to reach the target, which was set on 15% of their maximal grip force. If the cursor was held between 2 s and 4 s within the target they received a reward. Each hand was paired with one randomly assigned reward type (i.e., money for squeezing with the dominant hand, food for squeezing with the non-dominant hand). **(C)** During the Pavlovian conditioning, participants learned four associations between fractals and outcomes. Three outcomes were rewarding, whereas two of them were the same as during instrumental conditioning (upper two trials) and one was neutral (last trial). **(D)** During the Pavlovian-to-instrumental transfer, participants performed the same task as during instrumental conditioning but bimanually and under extinction. The same stimuli as in Pavlovian conditioning were shown in the background.

The whole experiment consisted of four different blocks: (1) the modified BDM auction; (2) the instrumental conditioning; (3) the Pavlovian conditioning; and (4) the PIT (Figure 1).

The BDM was used to match the subjective value of the monetary and food rewards to the subjective value of the social reward. These individually matched rewards were then used as reinforcing outcomes for all subsequent paradigms.

After the BDM procedure, participants underwent instrumental conditioning, where they learned two response-outcome contingencies. Participants performed separate effort tasks with their dominant and non-dominant hands and received a specific type of reward if the motor action was performed successfully (hand-reward pairings were counterbalanced across participants). For example, one person might learn that squeezing with the dominant hand lead to a monetary reward whilst squeezing with the non-dominant hand lead to a food reward, while another might learn that squeezing with the dominant hand lead to a social reward whilst squeezing with the non-dominant hand lead to a monetary reward.

Once participants had acquired the response-outcome contingencies, they underwent Pavlovian conditioning. In the Pavlovian conditioning trials, participants learned four different stimulus-outcome contingencies. Three stimuli were associated with one of the three reward-type outcomes (i.e., food, money or social reward) and a fourth stimulus was associated with a neutral outcome. Recall that for each participant, two of the three reward types were also previously presented during instrumental conditioning.

Lastly, we used PIT to investigate the influence of Pavlovian stimuli on instrumental responding. Here, the participants were instructed that they should squeeze the grip force handles bimanually, that they were free to distribute forces between hands and that they could stay inside a pre-defined target (set at 15% ± 2% MVC) for as long as they wanted to but maximally for the whole trial duration (6 s). In the end, this PIT paradigm allowed us to look at the force ratio between hands as a measure for the so-called “specific PIT” and at the time spent inside the target meaning how long they held the cursor at the required target force level or above as a measure for “general PIT”. A specific PIT is when a Pavlovian stimulus associated with a particular outcome selectively enhances the instrumental responding associated with that specific outcome (Corbit and Balleine, 2005; Cartoni et al., 2013). For example, participants squeeze more with their dominant hand when they observe the Pavlovian stimulus because both the stimulus and response have been paired with the monetary reward previously (i.e., via stimulus-outcome-response contingency). A general PIT effect is when a Pavlovian stimulus associated with a different reward engenders more instrumental responding for all outcomes (Corbit and Balleine, 2005; Cartoni et al., 2013). For example, participants spent more time in the target when they observe a stimulus, which was not paired with an instrumental action before.

### Modified Becker-DeGroot-Marschak Auction

The aim of this experimental block was to match the magnitude of the monetary and food reward to the subjective value of the social reward. Instead of bidding a certain amount of money for different outcomes as in the original BDM (Becker et al., 1964; Plassmann et al., 2007), we used physical effort as a common “currency” to quantify the subjective value of outcomes (Figure 1A). In a first step, participants had to hold a cursor for a certain number of seconds (randomized between 1 s and 10 s) in the target by squeezing the grip force handles bimanually with 50% of their MVC. After each trial, participants could recover for 10 s. This block was conducted in order to give the participants a better feeling for physical effort. During the actual BDM experiment, images of different reward magnitudes and reward types were presented (pseudo-randomized, 5 s) and after each reward, participants were required to bid the number of seconds (0–10) that they were willing to squeeze the grip force handles with 50% of their MVC to receive the presented reward. Then, the computer displayed (3 s) a random number between 0 and 10. If the random number was less than or equal to their bid, they had to exert a force of 50% of their MVC for the number of seconds equal to the random number, else they did not have to perform the squeezing. The social reward, which represented the social approval given by a smiling person in a thumbs-up pose (opposite gender than the participant and combined with joyful sound), was presented first. This was followed by randomly presented different amounts of Swiss Francs (0.1, 0.2, 0.5, 1.0, 1.2, 1.5, 2.0) and pieces of chocolate (1, 2, 4, 6, 8, 10, 12). Based on the participant’s bids, we matched the reward magnitude of the monetary and food rewards to the bid for the social reward.

### Instrumental Conditioning

Participants were instructed to either use their dominant or non-dominant hand to reach a target (15% MVC), which was displayed for 6 s on the screen (Figure 1B). To prevent participants from squeezing with both hands simultaneously, bimanual hand use was disabled by setting the cursor to zero when a certain amount of force (>0.5 N over 300 ms) was detected for both hands simultaneously. The participants’ goal of the block was to learn which reward they received when they used either their dominant or non-dominant hand to reach the target (e.g., dominant hand use leads to money, non-dominant hand use leads to food). The participants had to exert 15% MVC for 2–4 s (1/3 or 2/3 of the 6 s target display) to receive a reward. This rewarded time window was kept the same for both hands and over all trials. Participants received an outcome depending on which hand they used. The assignment of two out of the three matched rewards to either the dominant or non-dominant hand was random. An image of the specific reward in successful trials or a black screen in unsuccessful trials was displayed for 5 s. After 20 successful trials, the participants were asked, which reward they received after dominant or non-dominant hand use. If the answer was correct the instrumental conditioning was terminated, else the instrumental conditioning was repeated until they conducted another 20 successful trials. All participants answered the query correctly after a second block of instrumental conditioning (i.e., 40 trials) at the latest.

### Pavlovian Conditioning

In this experimental block, participants learned the associations between four Pavlovian stimuli (gray-scaled fractals) and four outcomes (Figure 1C). Three outcomes were positive (matched monetary, food and social rewards) and one was neutral (black screen). Therefore, participants experienced three rewarding outcomes during Pavlovian conditioning, while they experienced only two during instrumental conditioning. The associations between the Pavlovian stimuli and outcomes were pseudo-randomized across participants. A reinforcement schedule of 80% was administered (20% of trials were followed by the neutral outcome). The Pavlovian stimuli as well as the appropriate outcome were presented for 2 s. The inter-trial interval was 3 s. To increase the participant’s attention, they had to press the space bar every time a reward was displayed on the screen (although participants were told that pressing this space had no impact on reward outcome). After 60 randomized trials (15 per condition), the stimulus-outcome associations were tested. If the answers were incorrect, the procedure was repeated (120 trials in total) and otherwise the Pavlovian conditioning was terminated. Participants who did not learn the associations after 120 trials were excluded (see “Participants” Section).

### Pavlovian-to-Instrumental Transfer

Participants were instructed to use both hands simultaneously to reach the target (15% of the mean MVC of both hands) and that they will not receive any rewards anymore (i.e., under extinction). In each trial, we assessed whether the Pavlovian stimulus, shown full screen in the background for 6 s, influenced the extent to which participants distributed forces between hands (ratio between non-dominant and dominant force production) and how much time they spent in the target effort range (Figure 1D). A specific PIT predicts that the presentation of a specific stimulus (i.e., fractal A) will transfer to a specific response (i.e., stronger contribution of the dominant hand) because both the stimulus and the response have been paired with the same outcome during the instrumental and Pavlovian conditioning blocks. A general PIT effect would predict that the participants will spend more time in target when a stimulus was presented that has previously been associated with any reward. The inter-trial interval was 3 s. The PIT consisted of 80 randomly presented trials (20 trials per condition).

### Stimuli and Materials

We used four gray-scaled fractals as stimuli during the Pavlovian conditioning and PIT, which were matched to the same luminance and complexity (Willenbockel et al., 2010). Furthermore, we used images of coins (Swiss Francs), pieces of chocolate (Malteser^®^) and a smiling individual in a thumbs-up pose (opposite gender than the participant and combined with joyful sound) on a black background as reinforcing outcomes during the modified BDM auction, instrumental conditioning and Pavlovian conditioning (see e.g., Figure 1). The social rewards were meant to convey social approval and serve as a social reinforcer.

The experiment was programmed in LabView (National Instruments, Austin, TX, USA) and ran on a notebook (HP EliteBook 840, HP Company, Palo Alto, CA, USA). We used two custom-built grip force handles, printed by a 3D printer (Ultimaker^2^, Ultimaker B.V., Geldermalsen, Netherlands). Each grip force handle was 10 cm long and had an oval shape (diameters = 4.5 cm and 3.5 cm respectively). Two force sensors (FC22, Measurement Specialties, Fremont, CA, USA) were put inside the printed cases. Both handles were connected to a data acquisition box (NI USB-6009, National Instruments, Austin, TX, USA), which was then connected to the notebook and sampled with 200 Hz. A grip force handle was held in each hand, which allowed us to accurately measure the force produced by each hand during the modified BDM auction, instrumental conditioning and PIT.

### Analysis

Data was processed with a custom-made made script (Matlab 2013, MathWorks, Natick, MA, USA). We applied an adjusted boxplot criterion to correct for outliers within each condition and subject (Hubert and Vandervieren, 2008). Data points of subjects that differed more from the mean than ± 2.5 standard deviations were further considered as outliers and therefore, rejected from the analysis. Data was statically analyzed using mixed-effects models in SPSS 23 (IBM, Armonk, NY, USA). Mixed-effects models are more robust to non-normal distributed data and show a better fit for repeated measurements than conventional ANOVAs (Gueorguieva and Krystal, 2004; Gelman and Hill, 2007). PIT condition (hand one (*H1*), hand two (*H2*), no instrumental conditioning (*No IC*), *Neutral*) and reward type (*money, food, social*) were modeled as fixed effects depending on the analysis, and subjects were modeled as a random effect with random intercepts. We chose a compound symmetry covariance structure. Additionally, depending on the hypothesis, we added the subjective value as a covariate to the mixed-effects model. Bonferroni-corrected *post hoc* tests were applied if a significant main effect was detected in a mixed-effects model. We reported either Cohen’s *d* as a measure for effect size (*small d* = 0.20–0.49, *medium d* = 0.50–0.80, *large d* > 0.80; Cohen, 1988) or *r* (*small r* = 0.1–0.29, *medium r* = 0.3–0.49, *large r* > 0.5; Field, 2013). Furthermore, we used a Spearman’s correlation to show the relationship between subjective reward value and general PIT effect. In order to show equivalence between the different reward types, we conducted robust equivalence tests for paired samples (Yuen and Dixon, 1973; Schuirmann, 1981) using the software R (R Development Core Team, 2008). These tests make no assumptions about normality (Yuen and Dixon, 1973).

## Results

### Modified Becker-DeGroot-Marschak Auction

The aim of the BDM was to match monetary and food rewards to the same subjective reward value as the social reward. The subjective value of the social reward was rated in average as 6.26 (*range*: 2–10), which means that on average, participants bid 6.26 s of 50% grip force effort to receive a social reward. The corresponding matched monetary reward magnitude was on average 0.6 Swiss Francs (*standard deviation* = 0.6) and the corresponding matched food reward magnitude was on average four pieces of chocolate (*standard deviation* = 3).

To our knowledge, no previous study has used effort as a common currency for the BDM auction. We therefore analyzed the relationship between different amounts of Swiss Francs, as well as chocolate, and the subjective value quantified as physical effort. As expected, the higher the reward magnitude, the higher the subjective reward value, which represented an increased willingness to exert effort. This was supported by a strong positive correlation between reward magnitude and subjective value (monetary: *p* ≤ 0.001, *r* = 0.9837, *N* = 44; food: *p* ≤ 0.001, *r* = 0.9831, *N* = 42). Note that even though the monetary and food reward were not matched directly to each other, the corresponding amounts reflect the actual market value surprisingly well (0.15 Swiss Francs per piece of chocolate) confirming the face validity of our modified BDM.

### Instrumental and Pavlovian Conditioning

All participants successfully learned the associations during instrumental conditioning, with 89% (41 participants) needing 20 successful trials, and the others, 40 successful trials to learn the associations. Also all participants successfully learned the associations during Pavlovian conditioning, with 63% (29 participants) needing 60 trials and the rest 120 trials.

### General Pavlovian-to-Instrumental Transfer

**Figure 2:**
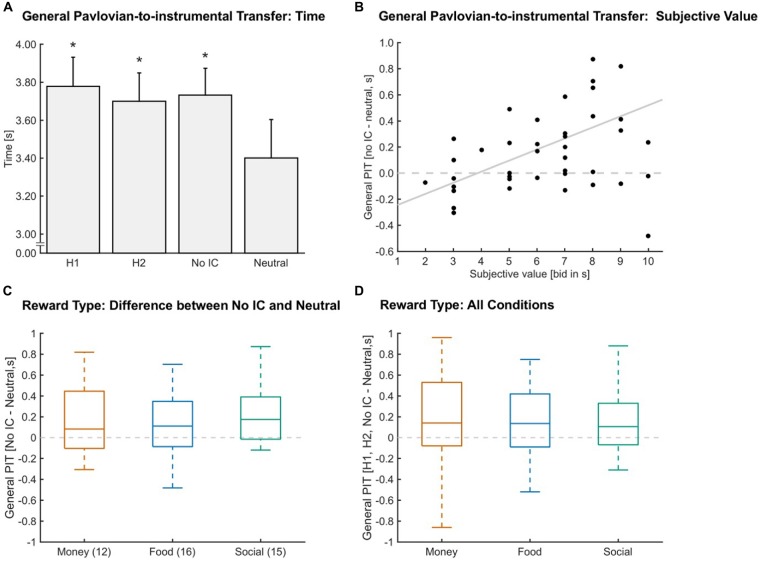
**General Pavlovian-to-instrumental transfer (PIT). (A)** A general PIT effect was found, given the significant increase in time in target for stimuli paired with a reward (Hand one 1 (*H1*), Hand two (*H2*), no instrumental conditioning (*No IC*)) compared to *Neutral* (*p ≤ 0.025, *N* = 44). Error bars indicate the standard error of the mean and asterisks indicate significant Bonferroni-corrected comparisons to *Neutral* (*p* ≤ 0.025). **(B)** General PIT defined as the difference between the *No IC—Neutral* condition significantly correlated with the subjective value (*r* = 0.389, *p* ≤ 0.025, *N* = 43). Values above the dashed horizontal line show a general PIT. The gray line indicates a linear fit. **(C)** The influence of reward type (money in orange, food in blue, social in green) on general PIT. Outliers according to a boxplot criterion are not depicted here because we applied a different criterion (mean ± 2.5 standard deviations). No statistical difference was found (*p* = 0.243, *N* = 43). Numbers in brackets indicate the number of participants for each reward type. The dashed gray line indicates no difference between *No IC* and *Neutral*. **(D)** General PIT collapsed across all instrumental conditions (*H1, H2, No IC*) in a within-subjects design depicted for each reward type (money in orange, food in blue, social in green). Outliers according to a boxplot criterion are not depicted here because we applied a different criterion (mean ± 2.5 standard deviations). No statistical difference was found (*p* = 0.077, *N* = 42). The dashed gray line indicates no difference between the rewarding conditions and *Neutral*.

To test for a general PIT effect, we compared the time spent in the target when each of the four different stimuli were presented in the background (see “Analysis” Section). The time spent inside the target range was used as a measure of the general PIT effect, because the criterion for receiving a reward in the instrumental task was based on time spent in the target range. Our mixed-effects analysis showed that previously learned stimulus associations significantly influenced the time participants spent inside the target force range (*F*_Condition (3,129)_ = 6.373, *p*_Condition_ ≤ 0.001, *N* = 44), such that participants spent significantly more time in the target when a reward-associated stimulus was presented compared to a neutral stimulus (*p*-value adjusted for multiple comparisons, *p*_H1-Neutral_ ≤ 0.025, *d*_H1-Neutral_ = 0.36, *p*_H2-Neutral_ ≤ 0.025, *d*_H2-Neutral_ = 0.26, *p*_No IC-Neutral_ ≤ 0.025, *d*_No IC-Neutral_ = 0.31; Figure 2A).

### The Impact of Subjective Value on General Pavlovian-to-Instrumental Transfer

The magnitude of the PIT effect increased in proportion to the subjective value of the rewards. When looking at the magnitude of PIT, defined as the difference between the *No IC* and *Neutral* condition, we observed that participants with higher general subjective values for the rewards also showed a higher general PIT (Figure 2B; *r* = 0.389, *p* ≤ 0.025, *N* = 43).

The magnitude of the general PIT furthermore significantly correlated with awareness scores (*r* = 0.608, *p* ≤ 0.001, *N* = 43). Thus, participants who pursued a conscious strategy during the PIT test showed a higher general PIT effect. A conscious strategy means that participants were able to report an explicit strategy relating the fractal identity to effort levels during debriefing after the PIT test. These conscious strategies were assessed separately from the quantification of contingency awareness after the instrumental and Pavlovian conditioning. All participants found to be unaware of the contingencies were excluded from the analyses (see “Participants” Section).

### The Impact of Different Reward Types on General Pavlovian-to-Instrumental Transfer

We examined the influence of reward type on general PIT in two ways. First, we used the rewards assigned to the *No IC* condition as a pure measure of Pavlovian influences because these rewards had no previous instrumental associations. Specifically, we computed the general PIT effect as the difference between the *No IC* and *Neutral* conditions. Each of the three reward types served as the *No IC* reward for a subset of the participants. *Money, Food*, and *Social* rewards were used in the *No IC* condition for 12, 16, and 15 participants, respectively. Our mixed-effects analysis suggested that all reward types were equally able to induce a general PIT effect (*F*_Reward Type (2,39)_ = 1.469, *p*_Reward Type_ = 0.243; *d*_Money-Food_ = 0.00, *d*_Money-Social_ = −0.24, *d*_Food-Social_ = −0.31; *N* = 43; Figure 2C). Nevertheless, general PIT was modulated by the individual level of motivation (added *Subjective Value* as a discrete covariate to the mixed-effects model), such that participants with a higher level of motivation, showed a stronger general PIT (*F*_Subjective Value (1,39)_ = 9.538, *p*_Subjective Value_ ≤ 0.025; *N* = 43).

Second, we tested the level of general PIT for all rewards collapsed across all preceding instrumental conditions (*H1, H2, No IC*) in order to achieve more statistical power in a within-subjects design. We computed the general PIT effects as the difference between each rewarding condition and the *Neutral* condition and assigned each general PIT effect to the corresponding reward type. We also found no statistical difference between reward types (*F*_Reward Type (2,82)_ = 2.65, *p*_Reward Type_ = 0.077; *d*_Money-Food_ = −0.09, *d*_Money-Social_ = 0.20, *d*_Food-Social_ = 0.31; *N* = 42; Figure 2D). Note that even though the statistics approach the traditional significance cutoff of 0.05 for the factor *Reward Type*, all effect sizes were small, which suggests only a minor practical relevance of reward type in PIT.

Subsequently, we used robust equivalence tests (Yuen and Dixon, 1973; Schuirmann, 1981) for paired samples to determine if the general PIT effects of different reward types are similar enough to be considered equivalent. The null hypothesis is described as follows: the difference is more than or equal to the predefined value of epsilon. We have chosen an epsilon value equal to one-half standard deviation of the general PIT effect across reward types (i.e., 250 ms). It is a common procedure to use one-half standard deviation to define the minimal important difference (Norman et al., 2003). We used a within-subjects design by assigning the general PIT effects of the three rewarding conditions (*H1, H2, No IC*) to the corresponding reward type. This substantially increases the power for demonstrating equivalence of the reward types. We detected a significant equivalence between reward types (*epsilon* = 0.250 s, *mean difference money − food* = 0.00, *p* ≤ 0.025, *mean difference money − social* = 0.04, *p* ≤ 0.025, *mean difference food − social* = 0.04, *p* ≤ 0.025, *N* = 42).

### Specific Pavlovian-to-Instrumental Transfer

**Figure 3:**
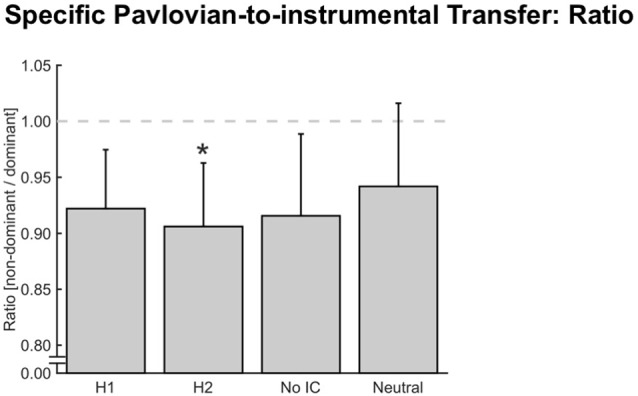
**Specific Pavlovian-to-instrumental transfer (PIT).** Error bars indicate the standard error of the mean and asterisks indicate significant Bonferroni-corrected comparisons to *Neutral* (*p* ≤ 0.025). Specific PIT looking at the force ratio between the non-dominant and dominant hand. A statistical difference was detected between hand two (*H2*) and *Neutral* condition (*p ≤ 0.025, *N* = 42). The dashed gray line indicates balanced hand use (ratio = 1).

In addition to an overall, reward-type independent response invigoration or motivation (i.e., general PIT), PIT paradigms have also been used to demonstrate outcome-specific PIT effects (i.e., specific PIT; Holmes et al., 2010; Cartoni et al., 2016). Our paradigm also allowed us to test for such specific PIT effects by analyzing how much force was assigned to each hand (dominant and non-dominant) when the four different stimuli were shown in the background. Here, we briefly remind the reader that specific outcomes (e.g., money, food, or social) were paired with dominant (*H1*) or non-dominant (*H2*) hand gripping. Therefore, in our bimanual grip force paradigm, a specific PIT effect would be present if the fractals used for the instrumental conditioning biased the force distribution between the dominant and non-dominant hand towards or away from the participant’s natural tendency to distribute force quasi-equally between hands in the bimanual setting. (i.e., *H1* should result in a higher force contribution of the dominant hand, and *H2* in a higher force contribution of the non-dominant hand). We calculated force ratios between the non-dominant and the dominant hand (Figure 3), which were all <1 indicating that a slight preference for squeezing with the dominant hand remained intact in all cases. Importantly, this preference was only moderately influenced by the presented fractal (*F*_Condition (3,123)_ = 3.26, *p*_Condition_ ≤ 0.025, *N* = 42). In particular, we found *less* non-dominant force contribution in *H2* than in *Neutral* (*p*_H2-Neutral_ ≤ 0.025, *p*-value Bonferroni adjusted for multiple comparisons, *d*_H2-Neutral_ = −0.12), which cannot be explained by the concept of specific PIT. Moreover, no other significant differences were found (*d*_H1-H2_ = 0.07, *d*_H1-Neutral_ = −0.08, *d*_No IC-Neutral_ = 0.08), suggesting that a specific PIT effect was not present when subjects responded with bimanual grip force (Figure 3).

We have also conducted an analysis that examines the force in specific PIT in more detail: (1) the timing (i.e., speed) with which each hand reached 5% or 15% MVC, and (2) the force ratio within the first 300 ms in the target range. We found no indication of a specific PIT effect in either case (*F*_timing 5% (3,135)_ = 1.36, *p*_timing 5%_ = 0.258, *N*_timing 5%_ = 46; *F*_timing 15% (3,135)_ = 1.68, *p*_timing 15%_ = 0.175, *N*_timing 15%_ = 46; *F*_ratio 300 ms (3,120)_ = 1.01, *p*_ratio 300 ms_ = 0.389, *N*_ratio 300 ms_ = 41).

Furthermore, we conducted another analysis investigating possible specific PIT effects looking at the time spent inside the target range. We calculated the time in target for trials where the fractal and hand dominance were congruent vs. incongruent to each other. A congruent trial was defined as a trial in which the participant contributed more force with the hand that was associated with the correct (i.e., rewarded) response for the currently displayed fractal during instrumental conditioning. Incongruent trials were the opposite and trials in which the fractal was not associated with reward for any response during instrumental conditioning were omitted. Note that this analysis collapses across cases in which *H1* and *H2* were congruent or incongruent in order to maximize power. We found no statistical difference in the time participants spent inside the target range between the congruent and incongruent trials (mixed-effects model, *F*_Condition (1,44)_ = 0.082, *p*_Condition_ = 0.775), and thus no indication of a specific PIT effect with this type of analysis either.

## Discussion

Our study was designed to investigate the influence of different reward types on behavior, when each reward type was calibrated to the same subjective value. A key feature of our study was the modified BDM using motor effort, instead of money, as a common currency. This allowed us to successfully match all the different types of reward to the same subjective value and then, conduct a well-controlled PIT experiment. Even though the monetary and food reward were not matched directly to each other, but rather calibrated independently to a specific subjective value, the inferred exchange rate between chocolate and money reflects the actual market value of a packet of Maltesers^®^ surprisingly well (0.15 Swiss Francs per piece of chocolate). Furthermore, previous studies using an incentive force task have shown that participants were willing to invest more effort for higher reward magnitudes (Pessiglione et al., 2007; Ziauddeen et al., 2012), which is in line with our results. Together, these two findings confirm the validity of our modified BDM.

We showed that individually matched monetary, food and social rewards successfully acted as appetitive reinforcers, such that stimuli associated with these rewards influenced instrumental behavior to a similar extent (Figure 2). Participants were willing to invest more effort when reward-associated cues were presented in the background compared to a neutral cue (Figure 2), which is in line with previous PIT research in humans (Bray et al., 2008; Talmi et al., 2008; Prévost et al., 2012; Watson et al., 2014, 2016; Garofalo and di Pellegrino, 2015). Furthermore, this general PIT effect was stronger, the higher the individual level of motivation, i.e., the higher the subjective reward value (Figure 2). Thus, we have shown that subjective reward value influences PIT in humans. To our knowledge, only one previous study in rodents has investigated the impact of reward magnitude on PIT and found that specific PIT was insensitive to reward magnitude (i.e., number of food pellets received during learning) by comparing the size of the PIT effect of a “low reward” to a “high reward” group (van den Bos et al., 2004). However, in the rodent study it is difficult to judge how strongly the subjective reward value differed between the “low reward” vs. the “high reward” group whereas subjective value was precisely measured here using the modified BDM. It is also possible that the subjective value only affects general PIT. Further research will be required to validate the influence of subjective value on PIT in a within-subjects design, which would have the advantage of being able to directly compare the influence of stimuli associated with different reward magnitudes on instrumental responding. Taken together, the findings from the present study support the hypothesis that the subjective reward value is an important determinant of how strongly instrumental responding is influenced by reward-associated stimuli.

We found no evidence that the reward type *per se* influences the general PIT effect. The absence of any differences between reward types in general PIT is in line with three different reward-processing frameworks (although the frameworks themselves make competing predictions in some cases).

First, Cartoni et al. (2013) link general PIT to utility so that a result of the action can be more or less valuable depending on the state of the agent (i.e., being hungry or sated). The motivational effect of general PIT is observed if the stimulus signals an unexpected, additional reward, which was previously not paired with that particular action during instrumental conditioning, increasing its utility. Thus, general PIT effects can be explained by the ability of reward-associated cues to also indicate the presence of other/additional rewards in the environment and thus, motivate the person to act as if they constitute an added value (Cartoni et al., 2013). Given that all rewards were value-matched in the present study; the added value was the same for every reward type.

Second, previous research has shown that general PIT effects depend mainly on the model-free system, which accumulates values through experience (i.e., temporal difference learning; Dolan and Dayan, 2013; Dayan and Berridge, 2014; Garbusow et al., 2014; Sebold et al., 2016). Model-free actions are by definition not executed with respect to the identity of the outcome. In the context of our study, a predominant engagement of the model-free system would result in a negligible influence of reward type on general PIT.

Third, the computation of subjective values for different reward types allows to map different reward types on a common scale to guide choices (Plassmann et al., 2007; Hare et al., 2008; Peters and Büchel, 2010; Rangel and Hare, 2010; Levy and Glimcher, 2011; Clithero and Rangel, 2014). This common-value framework also suggests that reward type itself would have a negligible influence on behavior as long as different reward types share the same subjective value.

Previous PIT studies have reported links between PIT effects and neural structures that are believed to support the computation of subjective reward values and reward learning. A large body of research in humans and animal models has identified a value-based choice and reward learning network consisting of orbitofrontal cortex (OFC), ventral tegmental area (VTA), substantia nigra (SN), amygdala, nucleus accumbens (NAcc) and vmPFC (for review see Ruff and Fehr, 2014). Dopaminergic neurons in VTA and SN have been shown to represent prediction errors, signals that are needed to update the anticipated value of rewards and stimuli (Schultz et al., 1997; Lak et al., 2014; Ruff and Fehr, 2014; Schultz, 2015). Additionally, the amygdala and OFC are thought to encode the anticipated value of stimuli (O’Doherty, 2004). Thus, it is not surprising that previous studies using a single reward type in humans have shown that the behavioral PIT effect was associated with increased activity in NAcc and amygdala (Talmi et al., 2008; Prévost et al., 2012; Garbusow et al., 2014, 2016). Similarly, links between single reward-type-PIT and NAcc and amygdala activity are well-established in rodents (Corbit et al., 2001; Hall et al., 2001; Holland and Gallagher, 2003; Corbit and Balleine, 2005; Holmes et al., 2010; McCue et al., 2014). Moreover, a recent rodent study demonstrated that a specific cortico-striatal circuit between medial PFC and NAcc is necessary to establish a successful PIT effect (Keistler et al., 2015). Neural signals in the medial PFC are believed to map all anticipated values and costs associated with different options onto a common scale to facilitate comparison and, ultimately, choosing between outcomes that potentially differ in the type of rewards they generate (Rushworth et al., 2011; Lin et al., 2012; Ruff and Fehr, 2014; Grueschow et al., 2015). Given our results showing that subjective-value matched rewards of three different types are equally effective in promoting PIT, we speculate that the medial PFC could also be a key neural structure modulating instrumental responding during PIT in humans by mapping different options on a common scale to make a final decision (Levy et al., 2012). Further neuroimaging studies are required to better understand the potential role of medial PFC on subcortical structures in PIT.

Although we found very clear general PIT effects, we did not see a specific PIT effect with any reward type. Based on previous research, there are a number of possible explanations: (1) We tested the participant’s responses using a transfer paradigm requiring bimanual responses while the instrumental conditioning was based on unimanual responses. There is only a partial overlap in neural control processes across unimanual and bimanual actions, which might diminish a transfer from the unimanual to the bimanual condition (Nozaki et al., 2006; Nozaki and Scott, 2009). In particular, healthy adults have a strong preference for distributing force quasi-symmetrical between the hands making the bimanual transfer condition relatively insensitive to detecting deviations from this strong, natural response tendency. (2) A shift to the non-dominant hand might have led to an increase in perceived effort and movement cost because the non-dominant hand is sometimes considered as noisier (Salimpour and Shadmehr, 2014), both of which might reduce the likelihood of engaging the non-dominant hand when it is not strictly necessary. (3) In contrast to the general PIT, none of the participants reported a conscious strategy for the specific PIT, which suggests that the distribution of force between hands was controlled unconsciously. Given that previous studies in humans have already shown the importance of contingency awareness in PIT (Talmi et al., 2008; Nadler et al., 2011; Lovibond et al., 2015), the unconscious control of bimanual force distribution may have diminished the specific PIT effect. Thus, the lack of specific PIT effects may be due to difficulties in establishing a transfer effect with this bimanual paradigm. Regardless of the limitations in terms of measuring specific PIT, our paradigm provides a clear demonstration of the influence of subjective-value on general PIT effects.

Despite its limitations, the methodology employed in our study lends itself to clinical investigations. Over the last few years, there has been converging evidence to suggest that reduced motivation to engage in social behavior may contribute to many social deficits observed in autism spectrum disorder (ASD; Scott-Van Zeeland et al., 2010; Delmonte et al., 2012; Richey et al., 2014; Barman et al., 2015). However, none of these studies have matched the subjective value of monetary and social stimuli, so it is not clear whether individuals with ASD have an issue with the subjective valuation network in general or if there is a specific social deficit. Testing participants with ASD and matched controls with an adapted BDM auction as we used in the present study, might help to better understand if the observed abnormalities in ASD could be explained by differences in the reward valuation system and/or in assigning incentive motivation to stimuli. Furthermore, we speculate that combining our behavioral methodologies with functional magnetic resonance imaging to investigate the role of the value-based network in the cross-sensitization of drugs (i.e., individuals suffering from alcohol dependence are on risk to also suffer from a nicotine dependence, Grant et al., 2004) could be a promising approach.

In conclusion, our study has demonstrated that stimuli of all reward types were able to act as appetitive reinforcers and influenced behavior, when matched on subjective reward value. The strength of the general PIT was modulated by subjective value (i.e., individuals who showed a stronger PIT effect rated the value of rewards more highly). These findings strengthen the hypotheses that the subjective value is crucial for how much reward-associated stimuli influence instrumental responding.

## Author Contributions

All authors conceived of and designed the experiment; RL programmed the experiment, analyzed the data, wrote the main manuscript text and prepared the figures; AH collected the data; all authors read, corrected and approved the final manuscript.

## Funding

This work was supported by the Eat2Move2Learn grant from the ETH Research Foundation.

## Conflict of Interest Statement

The authors declare that the research was conducted in the absence of any commercial or financial relationships that could be construed as a potential conflict of interest.

## References

[B1] Barman A. Richter S. Soch J. Deibele A. Richter A. Assmann A.. (2015). Gender-specific modulation of neural mechanisms underlying social reward processing by autism quotient. Soc. Cogn. Affect. Neurosci. 10, 1537–1547. 10.1093/scan/nsv044 25944965 PMC4631150

[B2] Becker G. M. DeGroot M. H. Marschak J. (1964). Measuring utility by a single-response sequential method. Behav. Sci. 9, 226–232. 10.1002/bs.3830090304 5888778

[B3] Bray S. Rangel A. Shimojo S. Balleine B. O’Doherty J. P. (2008). The neural mechanisms underlying the influence of pavlovian cues on human decision making. J. Neurosci. 28, 5861–5866. 10.1523/JNEUROSCI.0897-08.2008 18509047 PMC6670800

[B4] Cartoni E. Balleine B. Baldassarre G. (2016). Appetitive pavlovian-instrumental transfer: a review. Neurosci. Biobehav. Rev. 71, 829–848. 10.1016/j.neubiorev.2016.09.020 27693227

[B5] Cartoni E. Moretta T. Puglisi-Allegra S. Cabib S. Baldassarre G. (2015). The relationship between specific pavlovian instrumental transfer and instrumental reward probability. Front. Psychol. 6:1697. 10.3389/fpsyg.2015.01697 26635645 PMC4648073

[B6] Cartoni E. Puglisi-Allegra S. Baldassarre G. (2013). The three principles of action: a Pavlovian-instrumental transfer hypothesis. Front. Behav. Neurosci. 7:153. 10.3389/fnbeh.2013.00153 24312025 PMC3832805

[B7] Clithero J. A. Rangel A. (2014). Informatic parcellation of the network involved in the computation of subjective value. Soc. Cogn. Affect. Neurosci. 9, 1289–1302. 10.1093/scan/nst106 23887811 PMC4158359

[B8] Cohen J. D. (1988). Statistical power analysis for the behavioral-sciences. Percept. Mot. Skills 67, 1007–1007.

[B9] Corbit L. H. Balleine B. W. (2005). Double dissociation of basolateral and central amygdala lesions on the general and outcome-specific forms of pavlovian-instrumental transfer. J. Neurosci. 25, 962–970. 10.1523/JNEUROSCI.4507-04.2005 15673677 PMC6725628

[B10] Corbit L. H. Muir J. L. Balleine B. W. (2001). The role of the nucleus accumbens in instrumental conditioning: evidence of a functional dissociation between accumbens core and shell. J. Neurosci. 21, 3251–3260. 11312310 10.1523/JNEUROSCI.21-09-03251.2001 PMC6762583

[B11] Dayan P. Berridge K. C. (2014). Model-based and model-free Pavlovian reward learning: revaluation, revision and revelation. Cogn. Affect. Behav. Neurosci. 14, 473–492. 10.3758/s13415-014-0277-8 24647659 PMC4074442

[B12] Delmonte S. Balsters J. H. McGrath J. Fitzgerald J. Brennan S. Fagan A. J.. (2012). Social and monetary reward processing in autism spectrum disorders. Mol. Autism 3:7. 10.1186/2040-2392-3-7 23014171 PMC3499449

[B13] Dolan R. J. Dayan P. (2013). Goals and habits in the brain. Neuron 80, 312–325. 10.1016/j.neuron.2013.09.007 24139036 PMC3807793

[B14] Field A. (2013). Discovering Statistics Using IBM SPSS Statistics. London: SAGE Publications.

[B15] Garbusow M. Schad D. J. Sebold M. Friedel E. Bernhardt N. Koch S. P.. (2016). Pavlovian-to-instrumental transfer effects in the nucleus accumbens relate to relapse in alcohol dependence. Addict. Biol. 21, 719–731. 10.1111/adb.12243 25828702

[B16] Garbusow M. Schad D. J. Sommer C. Jünger E. Sebold M. Friedel E.. (2014). Pavlovian-to-instrumental transfer in alcohol dependence: a pilot study. Neuropsychobiology 70, 111–121. 10.1159/000363507 25359491

[B17] Garofalo S. di Pellegrino G. (2015). Individual differences in the influence of task-irrelevant Pavlovian cues on human behavior. Front. Behav. Neurosci. 9:163. 10.3389/fnbeh.2015.00163 26157371 PMC4478391

[B18] Gelman A. Hill J. (2007). Data Analysis Using Regression and Multilevel/Hierarchical Models. New York, NY: Cambridge University Press.

[B19] Grant B. F. Hasin D. S. Chou S. P. Stinson F. S. Dawson D. A. (2004). Nicotine dependence and psychiatric disorders in the United States: Results from the national epidemiologic survey on alcohol and related conditions. Arch. Gen. Psychiatry 61, 1107–1115. 10.1001/archpsyc.61.11.1107 15520358

[B20] Grueschow M. Polania R. Hare T. A. Ruff C. C. (2015). Automatic versus choice-dependent value representations in the human brain. Neuron 85, 874–885. 10.1016/j.neuron.2014.12.054 25640078

[B21] Gueorguieva R. Krystal J. H. (2004). Move over ANOVA: progress in analyzing repeated-measures data and its reflection in papers published in the archives of general psychiatry. Arch. Gen. Psychiatry 61, 310–317. 10.1001/archpsyc.61.3.310 14993119

[B22] Hall J. Parkinson J. A. Connor T. M. Dickinson A. Everitt B. J. (2001). Involvement of the central nucleus of the amygdala and nucleus accumbens core in mediating Pavlovian influences on instrumental behaviour. Eur. J. Neurosci. 13, 1984–1992. 10.1046/j.0953-816x.2001.01577.x 11403692

[B23] Hare T. A. O’Doherty J. Camerer C. F. Schultz W. Rangel A. (2008). Dissociating the role of the orbitofrontal cortex and the striatum in the computation of goal values and prediction errors. J. Neurosci. 28, 5623–5630. 10.1523/JNEUROSCI.1309-08.2008 18509023 PMC6670807

[B24] Holland P. C. Gallagher M. (2003). Double dissociation of the effects of lesions of basolateral and central amygdala on conditioned stimulus-potentiated feeding and Pavlovian-instrumental transfer. Eur. J. Neurosci. 17, 1680–1694. 10.1046/j.1460-9568.2003.02585.x 12752386

[B25] Holmes N. M. Marchand A. R. Coutureau E. (2010). Pavlovian to instrumental transfer: a neurobehavioural perspective. Neurosci. Biobehav. Rev. 34, 1277–1295. 10.1016/j.neubiorev.2010.03.007 20385164

[B26] Hubert M. Vandervieren E. (2008). An adjusted boxplot for skewed distributions. Comput. Stat. Data Anal. 52, 5186–5201. 10.1016/j.csda.2007.11.008

[B27] Huys Q. J. Cools R. Gölzer M. Friedel E. Heinz A. Dolan R. J.. (2011). Disentangling the roles of approach, activation and valence in instrumental and pavlovian responding. PLoS Comput. Biol. 7:e1002028. 10.1371/journal.pcbi.1002028 21556131 PMC3080848

[B28] Huys Q. J. Gölzer M. Friedel E. Heinz A. Cools R. Dayan P.. (2016). The specificity of Pavlovian regulation is associated with recovery from depression. Psychol. Med. 46, 1027–1035. 10.1017/s0033291715002597 26841896 PMC4825095

[B29] Keistler C. Barker J. M. Taylor J. R. (2015). Infralimbic prefrontal cortex interacts with nucleus accumbens shell to unmask expression of outcome-selective Pavlovian-to-instrumental transfer. Learn. Mem. 22, 509–513. 10.1101/lm.038810.115 26373829 PMC4579356

[B30] Lak A. Stauffer W. R. Schultz W. (2014). Dopamine prediction error responses integrate subjective value from different reward dimensions. Proc. Natl. Acad. Sci. U S A 111, 2343–2348. 10.1073/pnas.1321596111 24453218 PMC3926061

[B31] Levy D. J. Glimcher P. W. (2011). Comparing apples and oranges: using reward-specific and reward-general subjective value representation in the brain. J. Neurosci. 31, 14693–14707. 10.1523/JNEUROSCI.2218-11.2011 21994386 PMC3763520

[B32] Levy I. Rosenberg Belmaker L. Manson K. Tymula A. Glimcher P. W. (2012). Measuring the subjective value of risky and ambiguous options using experimental economics and functional MRI methods. J. Vis. Exp. 67:e3724. 10.3791/3724 23022992 PMC3490235

[B33] Lewis A. H. Niznikiewicz M. A. Delamater A. R. Delgado M. R. (2013). Avoidance-based human Pavlovian-to-instrumental transfer. Eur. J. Neurosci. 38, 3740–3748. 10.1111/ejn.12377 24118624 PMC3865081

[B34] Lin A. Adolphs R. Rangel A. (2012). Social and monetary reward learning engage overlapping neural substrates. Soc. Cogn. Affect. Neurosci. 7, 274–281. 10.1093/scan/nsr006 21427193 PMC3304477

[B35] Lovibond P. F. Satkunarajah M. Colagiuri B. (2015). Extinction can reduce the impact of reward cues on reward-seeking behavior. Behav. Ther. 46, 432–438. 10.1016/j.beth.2015.03.005 26163708

[B36] McCue M. G. LeDoux J. E. Cain C. K. (2014). Medial amygdala lesions selectively block aversive pavlovian-instrumental transfer in rats. Front. Behav. Neurosci. 8:329. 10.3389/fnbeh.2014.00329 25278858 PMC4166994

[B37] Nadler N. Delgado M. R. Delamater A. R. (2011). Pavlovian to instrumental transfer of control in a human learning task. Emotion 11, 1112–1123. 10.1037/a0022760 21534664 PMC3183152

[B38] Norman G. R. Sloan J. A. Wyrwich K. W. (2003). Interpretation of changes in health-related quality of life: the remarkable universality of half a standard deviation. Med. Care 41, 582–592. 10.1097/01.mlr.0000062554.74615.4c 12719681

[B40] Nozaki D. Kurtzer I. Scott S. H. (2006). Limited transfer of learning between unimanual and bimanual skills within the same limb. Nat. Neurosci. 9, 1364–1366. 10.1038/nn1785 17028583

[B39] Nozaki D. Scott S. H. (2009). Multi-compartment model can explain partial transfer of learning within the same limb between unimanual and bimanual reaching. Exp. Brain Res. 194, 451–463. 10.1007/s00221-009-1720-x 19205679

[B41] O’Doherty J. P. (2004). Reward representations and reward-related learning in the human brain: insights from neuroimaging. Curr. Opin. Neurobiol. 14, 769–776. 10.1016/j.conb.2004.10.016 15582382

[B42] Oldfield R. C. (1971). The assessment and analysis of handedness: the Edinburgh inventory. Neuropsychologia 9, 97–113. 10.1016/0028-3932(71)90067-4 5146491

[B43] Pessiglione M. Petrovic P. Daunizeau J. Palminteri S. Dolan R. J. Frith C. D. (2008). Subliminal instrumental conditioning demonstrated in the human brain. Neuron 59, 561–567. 10.1016/j.neuron.2008.07.005 18760693 PMC2572733

[B44] Pessiglione M. Schmidt L. Draganski B. Kalisch R. Lau H. Dolan R. J.. (2007). How the brain translates money into force: a neuroimaging study of subliminal motivation. Science 316, 904–906. 10.1126/science.1140459 17431137 PMC2631941

[B45] Peters J. Büchel C. (2010). Neural representations of subjective reward value. Behav. Brain Res. 213, 135–141. 10.1016/j.bbr.2010.04.031 20420859

[B46] Plassmann H. O’Doherty J. Rangel A. (2007). Orbitofrontal cortex encodes willingness to pay in everyday economic transactions. J. Neurosci. 27, 9984–9988. 10.1523/JNEUROSCI.2131-07.2007 17855612 PMC6672655

[B47] Prévost C. Liljeholm M. Tyszka J. M. O’Doherty J. P. (2012). Neural correlates of specific and general Pavlovian-to-Instrumental transfer within human amygdalar subregions: a high-resolution fMRI study. J. Neurosci. 32, 8383–8390. 10.1523/JNEUROSCI.6237-11.2012 22699918 PMC6703659

[B48] Quail S. L. Morris R. W. Balleine B. W. (2017). Stress associated changes in Pavlovian-instrumental transfer in humans. Q. J. Exp. Psychol. (Hove) 70, 675–685. 10.1080/17470218.2016.1149198 26878746

[B50] Rangel A. Hare T. (2010). Neural computations associated with goal-directed choice. Curr. Opin. Neurobiol. 20, 262–270. 10.1016/j.conb.2010.03.001 20338744

[B49] R Development Core Team (2008). R: A Language and Environment for Statistical Computing. Vienna, Austria: R Foundation for Statistical Computing.

[B51] Richey J. A. Rittenberg A. Hughes L. Damiano C. R. Sabatino A. Miller S.. (2014). Common and distinct neural features of social and non-social reward processing in autism and social anxiety disorder. Soc. Cogn. Affect. Neurosci. 9, 367–377. 10.1093/scan/nss146 23223206 PMC3980795

[B52] Ruff C. C. Fehr E. (2014). The neurobiology of rewards and values in social decision making. Nat. Rev. Neurosci. 15, 549–562. 10.1038/nrn3776 24986556

[B53] Rushworth M. F. Noonan M. P. Boorman E. D. Walton M. E. Behrens T. E. (2011). Frontal cortex and reward-guided learning and decision-making. Neuron 70, 1054–1069. 10.1016/j.neuron.2011.05.014 21689594

[B54] Salimpour Y. Shadmehr R. (2014). Motor costs and the coordination of the two arms. J. Neurosci. 34, 1806–1818. 10.1523/JNEUROSCI.3095-13.2014 24478362 PMC3905146

[B55] Saxe R. (2006). Uniquely human social cognition. Curr. Opin. Neurobiol. 16, 235–239. 10.1016/j.conb.2006.03.001 16546372

[B56] Schuirmann D. L. (1981). On hypothesis-testing to determine if the mean of a normal-distribution is contained in a known interval. Biometrics 37, 617–617.

[B57] Schultz W. (2015). Neuronal reward and decision signals: from theories to data. Physiol. Rev. 95, 853–951. 10.1152/physrev.00023.2014 26109341 PMC4491543

[B58] Schultz W. Dayan P. Montague P. R. (1997). A neural substrate of prediction and reward. Science 275, 1593–1599. 10.1126/science.275.5306.1593 9054347

[B59] Scott-Van Zeeland A. A. Dapretto M. Ghahremani D. G. Poldrack R. A. Bookheimer S. Y. (2010). Reward processing in autism. Autism Res. 3, 53–67. 10.1002/aur.122 20437601 PMC3076289

[B60] Sebold M. Schad D. J. Nebe S. Garbusow M. Jünger E. Kroemer N. B.. (2016). Don’t think, just feel the music: individuals with strong pavlovian-to-instrumental transfer effects rely less on model-based reinforcement learning. J. Cogn. Neurosci. 28, 985–995. 10.1162/jocn_a_00945 26942321

[B61] Sescousse G. Caldú X. Segura B. Dreher J. C. (2013). Processing of primary and secondary rewards: a quantitative meta-analysis and review of human functional neuroimaging studies. Neurosci. Biobehav. Rev. 37, 681–696. 10.1016/j.neubiorev.2013.02.002 23415703

[B62] Talmi D. Seymour B. Dayan P. Dolan R. J. (2008). Human pavlovian-instrumental transfer. J. Neurosci. 28, 360–368. 10.1523/JNEUROSCI.4028-07.2008 18184778 PMC2636904

[B63] van den Bos R. van der Harst J. Vijftigschild N. Spruijt B. van Luijtelaar G. Maes R. (2004). On the relationship between anticipatory behaviour in a Pavlovian paradigm and Pavlovian-to-instrumental transfer in rats *(Rattus norvegicus)*. Behav. Brain Res. 153, 397–408. 10.1016/j.bbr.2003.12.017 15265635

[B64] Watson P. Wiers R. W. Hommel B. de Wit S. (2014). Working for food you don’t desire. Cues interfere with goal-directed food-seeking. Appetite 79, 139–148. 10.1016/j.appet.2014.04.005 24743030

[B65] Watson P. Wiers R. W. Hommel B. Ridderinkhof K. R. de Wit S. (2016). An associative account of how the obesogenic environment biases adolescents’ food choices. Appetite 96, 560–571. 10.1016/j.appet.2015.10.008 26482282

[B66] Willenbockel V. Sadr J. Fiset D. Horne G. O. Gosselin F. Tanaka J. W. (2010). Controlling low-level image properties: the SHINE toolbox. Behav. Res. Methods 42, 671–684. 10.3758/BRM.42.3.671 20805589

[B67] World Medical Association. (2013). World Medical Association Declaration of Helsinki: Ethical principles for medical research involving human subjects. JAMA 310, 2191–2194. 10.1001/jama.2013.281053 24141714

[B68] Yuen K. K. Dixon W. J. (1973). The approximate behavior and performance of two-sample trimmed T. Biometrika 60, 369–374. 10.2307/2334550

[B69] Ziauddeen H. Subramaniam N. Gaillard R. Burke L. K. Farooqi I. S. Fletcher P. C. (2012). Food images engage subliminal motivation to seek food. Int. J. Obes. 36, 1245–1247. 10.1038/ijo.2011.239 22143617 PMC3438467

